# Is the use of high correlated color temperature light at night related to delay of sleep timing in university students? A cross-country study in Japan and China

**DOI:** 10.1186/s40101-021-00257-x

**Published:** 2021-06-08

**Authors:** Shigekazu Higuchi, Yandan Lin, Jingjing Qiu, Yichi Zhang, Michihiro Ohashi, Sang-il Lee, Shingo Kitamura, Akira Yasukouchi

**Affiliations:** 1 Department of Human Science, Faculty of Design, Kyushu University, 4-9-1 Shiobaru, Minamiku, Fukuoka, 815-8540 Japan; 2 Institute for Electric Light Sources, Fudan University, Shanghai, 200433 China; 3 Department of Kansei Science, Graduate School of Integrated Frontier Sciences, Kyushu University, 4-9-1 Shiobaru, Minamiku, Fukuoka, 815-8540 Japan; 4 Laboratory of Environmental Ergonomics, Faculty of Engineering, Hokkaido University, Kita 8, Nishi 5, Kita-ku, Sapporo, Hokkaido 060-0808 Japan; 5 Department of Sleep-Wake Disorders, National Institute of Mental Health, National Center of Neurology and Psychiatry, 4-1-1, Ogawa-Higashi, Kodaira, Tokyo, 187-8553 Japan

**Keywords:** Circadian rhythm, Sleep, Blue light, Social jetlag, University students, International comparison

## Abstract

**Background:**

Blue-enriched white light at night has the potential to delay the circadian rhythm in daily life. This study was conducted to determine whether the use of high correlated color temperature (CCT) light at home at night is associated with delay of sleep timing in university students.

**Methods:**

The survey was conducted in 2014–2015 in 447 university students in Japan and 327 students in China. Habitual sleep timing and type of CCT light at home were investigated by using a self-administered questionnaire. The Japanese students were significantly later than the Chinese students in bedtime, wake time, and midpoint of sleep. They were asked whether the lighting in the room where they spend most of their time at night was closer to warm color (low CCT) or daylight color (high CCT). The amount of light exposure level during daily life was measured for at least 1 week by the use of a light sensor in 60 students in each country.

**Results:**

The percentages of participants who used high CCT lighting at night were 61.6% for Japanese students and 80.8% for Chinese students. Bedtime and sleep onset time on school days and free days were significantly later in the high CCT group than in the low CCT group in Japan. The midpoint of sleep in the high CCT group was significantly later than that in the low CCT group on free days but not on school days. On the other hand, none of the sleep measurements on school days and free days were significantly different between the high CCT and low CCT groups in China. Illuminance level of light exposure during the night was significantly higher in Japanese than in Chinese, but that in the morning was significantly higher in China than in Japan.

**Conclusions:**

The use of high CCT light at night is associated with delay of sleep timing in Japanese university students but not in Chinese university students. The effects of light at night on sleep timing and circadian rhythm may be complicated by other lifestyle factors depending on the country.

**Supplementary Information:**

The online version contains supplementary material available at 10.1186/s40101-021-00257-x.

## Background

Light is a strong synchronizing agent (zeitgeber) for the circadian system. Light exposure in the morning has an important role in resetting the circadian rhythm to a 24-h cycle in humans. On the other hand, evening light exposure delays the phase of circadian rhythms. This relationship between the timing of light exposure and phase shift of circadian rhythms is known as a phase-response curve [1, 2]. In addition to the effect on the circadian rhythm, light at night has an impact on suppression of melatonin secretion [3] and an increase in arousal level [4]. These physiological and endocrinological effects are known as non-visual effects of light and/or non-image-forming effects of light [5–7]. In modern society, most people spend the night safely and comfortably thanks to artificial lighting at night. However, excessive exposure to light at night has negative effects on human sleep and circadian rhythm [8].

The non-visual effects of light depend on the intensity of light, the spectrum of light, and the duration of exposure. The light intensity of even general home lighting is known to affect circadian rhythms and melatonin secretion [9, 10]. Regarding the light spectrum, blue light is known to have a strong effect on melatonin suppression and phase shift of the circadian rhythm [11–13]. Intrinsically photosensitive retinal ganglion cells (ipRGCs), which contain a photopigment called melanopsin, have an important role in the action of blue light [14, 15]. The action of blue light is observed in blue-enriched polychromatic light, which is called high correlated color temperature (CCT) light. High CCT light has greater effects than low CCT light on arousal level and melatonin suppression [16–18]. The physiological effects of high CCT of a fluorescent lamp were reported even before the discovery of ipRGCs [19–22]. In recent years, similar results have been obtained by exposure to LED lighting [23–25].

However, many of those studies were conducted in a laboratory, and there have been few studies in which the influence of CCT of home lighting in real life was examined. We previously reported that there were significant correlations between CCT of lighting at home and phase of the circadian rhythm in middle-aged adults and primary school children in Japan [26]. The results of that study suggested that high CCT light at home might be a cause of the delay of the circadian phase in daily life. However, that study had a small sample size and there has been no study conducted in a large population. This study was conducted in a large number of university students. It is known that university students have the most night-typed sleep pattern [27].

Furthermore, the survey was conducted in Japan and China. Our previous study conducted in Japan showed that the CCT of lighting used in the home correlates with circadian rhythms [26]. However, sleep habits and circadian rhythms are influenced not only by the light environment but also by the lifestyle and social environment [28]. It is possible that the CCT of artificial lighting at night has an effect on sleep only in Japan. To generalize the effect of CCT on sleep in real life, it was necessary to research outside Japan. Therefore, this study also included Chinese university students living in a social environment different from that in Japan. We chose a location where the latitude of the study site, Shanghai in China, is almost the same as that of Fukuoka in Japan, and the times of sunrise and sunset are also almost the same.

The purpose of this study was to determine the differences between light environments in China and Japan and to clarify the relationship between light environments at night and sleep habits in university students in Japan and China.

## Methods

### Participants

The study was conducted in mid-December 2014 and mid-January 2015. The year-end and New Year holidays were excluded from the survey period. The participants included 470 Japanese university students and 397 Chinese university students. All participants provided informed written consent. The study was approved by the Institutional Ethics Committee of Kyushu University; the protocol and all the procedures were in agreement with the Declaration of Helsinki. After excluding participants whose data included typographical errors or for whom data were missing, we finally analyzed data for 447 Japanese students (average age of 21.5 ± 1.6 years) and 327 Chinese students (average age of 23.3 ± 2.7 years). The ratio of males in the Japanese students (62.8%) was significantly higher than that in the Chinese students (53.4%).

The survey was conducted in Fukuoka City in Japan and Shanghai City in China. The latitudes of Fukuoka and Shanghai are 33° 36′ north latitude and 31° 11′ north latitude, respectively. The time difference between Japan and China is 1 h. The local times at sunrise on December 1st in Fukuoka and Shanghai were 7:04 and 6:34, respectively, and the local times at sunset were 17:10 and 16:51, respectively.

### Measurements of sleep habits

We investigated the habitual bedtime (lights off time), subjective sleep latency, and wake time on school days and free days using a self-administered questionnaire. The sleep onset time was calculated by adding the sleep latency to the bedtime, and the sleep period time was calculated by the time from the sleep onset time to the wake time. The sleep midpoint was calculated by adding 1/2 of the sleep period time to the sleep onset time. Social jet lag was calculated as the absolute difference between midpoint of sleep on free days and midpoint on school days [29].

Table 1 shows the sleep habits of the university students in Japan and China in the present study. After adjustments by sex and age, on both school days and free days, the Japanese students were significantly later than the Chinese students in all measurements including bedtime, sleep onset time, wake time, and midpoint of sleep (*p* < 0.001). Sleep period time was also significantly shorter in Japanese students than in Chinese students  (*p* < 0.001). Subjective sleep latency   was not significantly different between the two groups. Social jet lag was significantly larger in Japanese students than in Chinese students (*p* < 0.001).

**Table 1:** Demographic data and sleep habits in university students in Japan and China

	Japanese (*n* = 447)	Chinese (*n* = 327)	
Mean age (years)	21.5 (1.6)	23.3 (2.7)	**
Sex, %male	62.8	53.4	**
**Sleep habits** (school days)			
Bedtime	01:09 (1:01)	23:58 (0:43)	***
Sleep latency (min)	15.1 (11.3)	16.1 (11.7)	n.s.
Sleep onset time	01:24 (1:02)	00:15 (0:46)	***
Wake time	08:01 (1:11)	07:42 (0:49)	***
Midpoint of sleep	04:43 (0:57)	3:58 (0:39)	***
Sleep period time (h)	6.62 (1.13)	7.46 (0.90)	***
**Sleep habits** (free days)			
Bedtime	01:37 (1:12)	00:22 (0:51)	***
Sleep latency (min)	16.0 (13.0)	14.9 (11.7)	n.s.
Sleep onset time	01:52 (1:14)	00:38 (0:52)	***
Wake time	09:35 (1:25)	08:46 (1:01)	***
Midpoint of sleep	05:45 (1:11)	4:42 (0:49)	***
Sleep period time (h)	7.70 (1.25)	8.15 (0.95)	***
Social jet lag (h)	1.04 (0.82)	0.73 (0.56)	***

Values are means and SD

Adjusted by age and sex **p < 0.01, ***p < 0.001, *n.s* not significant

### Measurements of light environments

To the best of our knowledge, this study is the first study in which the type of CCT light used at home was investigated for a large sample using a questionnaire. Therefore, we originally made a simple two-choice question. The participants were asked whether the lighting in the room where they spend most of their time at night was closer to warm color (low CCT) or daylight color (high CCT). Color photographs were used to help participants make accurate selections. The participants looked at two photographs of lighting in the questionnaire and chose one of them. The photographs used in the questionnaire in this study are not allowed to be shown here due to copyright issues, but almost the same pictures are shown as a supplemental file (see Additional file 1).

In addition, we asked 60 Japanese and 60 Chinese subjects to wear a pendant-type illuminance sensor (HOBO by Onset UA-002-08) and illuminance was continuously measured for about 2 weeks at 2-min intervals. After exclusion of subjects due to missing data, data for 54 Japanese students (22.5 ± 2.2 years old, %male = 51.9) and 58 Chinese students (22.6 ± 2.2 years old, %male = 50.4) were used for analysis. Sleep timing was significantly later in Japanese students than in Chinese students (Additional file 2).

The resolution of illuminance was about 10 lx. The subjects were instructed to always wear the sensor while awake, except for exercise and bathing, and keep it at the bedside while sleeping. First, the average illuminance was calculated for each hour to see the change over a period of 24 h. In another way, illuminance level was calculated on the basis of habitual bedtime. Average illuminance level for 2 h before habitual bedtime was used as a representative illuminance level before bedtime at home at night for each participant. Data with 0 lx before habitual bedtime were excluded from the calculations. Due to the limited number of measurement days on free days, the data for free days and  school days were not separated.

### Statistical analysis

The chi-square test was used for comparison of the percentages of Japanese and Chinese students who use high CCT lighting. As for the illuminance level, a t-test was conducted after logarithm transformation. A comparison of sleep measurements between CCT lighting conditions was made by adjusting age and sex using a linear mixed model since sleep timing and sleep hours are affected by sex and age [27]. Pearson’s correlation analysis was performed for illuminance level and sleep measurements.

## Results

**Fig. 1:**
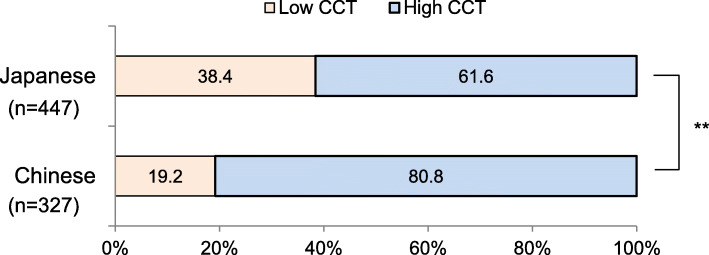
Percentages of users of high CCT light and low CCT light at home at night in Japanese and Chinese university students. **p < 0.01

**Fig. 2:**
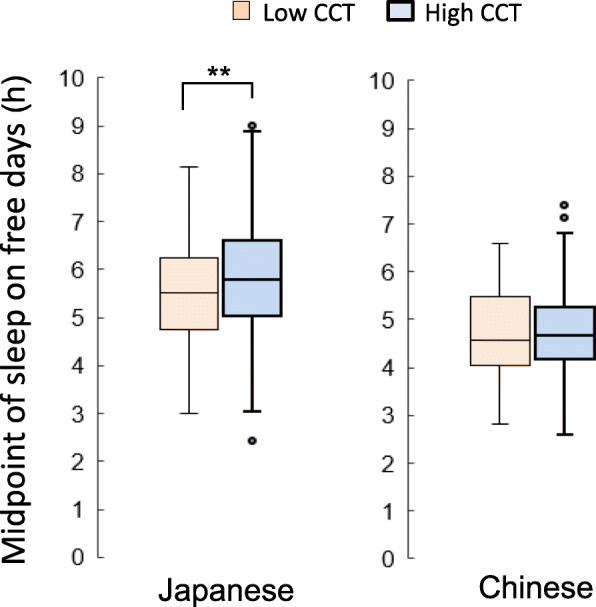
Box plots of the midpoints of sleep on free days in the high CCT group and the low CCT group in Japanese (left) and Chinese (right). **p < 0.01

The percentages of participants who used high CCT lighting at night were 61.6% for Japanese students and 80.8% for Chinese students (Fig. 1). The percentage of participants who used high CCT lighting was significantly higher for Chinese students (chi-square test, *p* < 0.05). Sleep habits were compared in the high CCT group and the low CCT group for both Japanese students and Chinese students after adjusting for sex and age. In the Japanese students, bedtime and sleep onset time in the high CCT group were significantly later than those in the low CCT group on school days (*p* < 0.05) and free days (*p* < 0.01) (Table 2). Also, the midpoint of sleep in the high CCT group was significantly later than that in the low CCT group on free days (*p* < 0.01) (Fig. 2). The wake time on flee days tended to be later (*p* = 0.052) and social jet lag (*p* = 0.051) tended to be larger in the high CCT group than in the low CCT group. On the other hand, in the Chinese students, there was no significant difference in sleep measurements between the high CCT group and the low CCT group on school days or free days (Table 3).

**Table 2:** Sleep habits on school days and free days in university students in Japan

Japanese	High CCT (*n* = 275)	Low CCT (*n* = 172)	*P* value
**Sleep habits** (school days)			
Bedtime	01:13 (0:58)	01:00 (1:04)	0.029 *
Sleep latency (min)	15.5 (11.8)	15.1 (10.7)	0.668
Sleep onset time	01:29 (1:00)	01:15 (1:05)	0.026 *
Wake time	08:02 (1:13)	07:59 (1:10)	0.476
Midpoint of sleep	04:45 (0:57)	4:37 (0:59)	0.100
Sleep period time (h)	6.55 (1.14)	6.74 (1.11)	0.186
**Sleep habits** (free days)			
Bedtime	01:45 (1:13)	01:25 (1:11)	0.003 **
Sleep latency (min)	16.1 (13.3)	16.1 (12.6)	0.949
Sleep onset time	02:02 (1:16)	01:41 (1:11)	0.003 **
Wake time	09:41 (1:27)	09:26 (1:20)	0.052
Midpoint of sleep	05:51 (1:11)	05:33 (1:08)	0.006 **
Sleep period time (h)	7.65 (1.33)	7.74 (1.12)	0.495
Social jet lag (h)	1.09 (0.84)	0.94 (0.78)	0.051

Values are means and SD.

Adjusted by age and sex *p < 0.05, **p < 0.01

**Table 3:** Sleep habits on school days and free days in university students in China

Chinese	High CCT (*n* = 271)	Low CCT (*n* = 56)	*P* value
**Sleep habits** (school days)			
Bed time Bedtime	23:58 (0:43)	00:01 (0:46)	0.790
Sleep latency (min)	16.1 (10.4)	16.2 (16.9)	0.891
Sleep onset time	00:14 (0:45)	00:17 (0:48)	0.770
Wake time	07:41 (0:48)	07:45 (0:55)	0.442
Midpoint of sleep	03:58 (0:38)	04:01 (0:43)	0.510
Sleep period time (h)	7.45 (0.88)	7.48 (0.92)	0.645
**Sleep habits** (free days)			
Bedtime	00:23 (0:50)	00:22 (0:58)	0.803
Sleep latency (min)	14.7 (10.3)	15.7 (16.9)	0.561
Sleep onset time	00:38 (0:51)	00:37 (0:57)	0.909
Wake time	08:48 (1:02)	08:42 (0:59)	0.508
Midpoint of sleep	04:43 (0:49)	04:40 (0:53)	0.633
Sleep period time (h)	8.16 (0.99)	8.08 (0.75)	0.544
Social jet lag (h)	0.79 (0.54)	0.75 (0.67)	0.153

Values are means and SD

Adjusted by age and sex

**Fig. 3:**
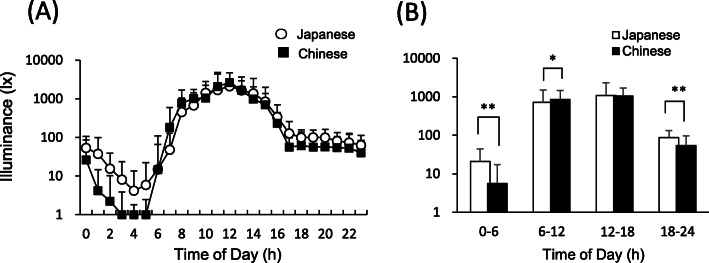
Changes in the illuminance level that the participants were exposed to for 24 h in Japan (*n* = 54) and China (*n* = 58) (**A**). The illuminance on the vertical axis is displayed in logarithm. Average illuminance levels from midnight to early morning (0:00–6:00), in the morning (6:00–12:00), in the afternoon (12:00–18:00), and in the evening (18:00–24:00) (**B**). Data are shown as averages and standard deviations. (*p < 0.05, **p < 0.01)

Figure 3 shows the changes in illuminance level that the participants were exposed to for 24 h. The average illuminance in the evening (18:00–24:00) was significantly higher for the Japanese students than for the Chinese students (t = 5.938, *p* < 0.001). The average illuminance was significantly higher for the Japanese students than for the Chinese students during the period from midnight to early morning (0:00–6:00) including sleeping time (t = 7.573, *p* < 0.001). On the other hand, the average illuminance in the morning (6:00–12:00) was significantly higher for the Chinese students than for the Japanese students (t = − 2.038, *p* < 0.05). There was no significant difference between illuminance levels in the afternoon (12:00–18:00) for the Japanese students and Chinese students. The average illuminance level for 2 h before habitual bedtime was significantly higher for the Japanese students (74.8 lx ± 56.5 lx) than for the Chinese students (59.9 lx ± 64.8 lx) (t = 7.573, *p* < 0.01). Correlation analysis was conducted for illuminance level for 2 h before habitual bedtime at night and sleep habits. There was no significant correlation between illuminance level and any of the sleep measurements for both the Japanese students and Chinese students.

## Discussion

The results of investigation of the relation between the CCT of lighting and sleep habits in each country showed that bedtime and sleep onset time of the students who used high CCT lighting was significantly later on school days and free days for the Japanese students. Previous laboratory studies showed that high CCT lighting (blue-enriched white lighting) has a greater impact on arousal level and melatonin suppression [16–18]. The present study supported the results of previous studies conducted in a laboratory, and the association between use of high CCT lighting at night and delay of sleep timing was confirmed in the real world in a large sample of Japanese students. The delay in sleep timing for users of high CCT lighting in Japan is thought to be caused by an increase in alertness and delay of the circadian rhythm induced by exposure to blue-enriched light before bedtime.

The difference between sleep habits of students in the high CCT group and the low CCT group in Japan was more pronounced on free days than on school days. The midpoint of sleep on free days was significantly later in the high CCT group than in the low CCT group (Fig. 2). Also, wake-up time on free days tended to be delayed in the high CCT group. Therefore, the delay in the midpoint of sleep on free days might be caused not only by the delay in bedtime but also by the delay in wake-up time. It is known that a free day without social restriction tends to reflect individual chronotypes [30]. Furthermore, in this study, social jet lag tended to increase with high CCT. Social jet lag is known to be larger in night type persons [31, 32]. From the above, this result suggests that the use of high CCT at night is associated with the night type chronotype. It has been reported that social jet lag is associated with obesity [29], depressive state [33], academic performance [34], and menstrual symptoms [35]. Further analysis of the associations between light environments, sleep, and health status is needed.

In the present study, significant differences in sleep habits between lighting conditions were found in Japanese students but not in Chinese students. One of the reasons for this difference may be the difference in illuminance level. Illuminance level after sunset for the Japanese students was significantly higher than that for the Chinese students. It is known that the effects of light also depend on the illuminance [4]. Therefore, it is possible that the effect of the CCT of light on sleep habits was small under the condition of the lower illuminance level in China. In contrast to light at night, the amount of light exposure in the morning was significantly larger in the Chinese students than in Japanese students. This suggests that the reset of the circadian rhythm by morning light exposure in China might prevent bedtime delay even though high CCT lighting is used at night.

Furthermore, the effects of light at night on sleep timing may be complicated by other lifestyle factors depending on the country. For example, in this study, about 80% of the Chinese students lived in dormitories and about 70% of the Japanese students lived alone. Chinese students lived with quite regular timetables for classes. Weak social pressure for a sleep/wake schedule is considered to be related to the delay of sleep pattern in Japanese students. This means that Japanese students are exposed to artificial lighting for a longer period of time before going to bed, which might be a cause of the delay of circadian rhythm as shown in a previous study [36]. In other words, relatively strong social pressure for sleep/wake timing in Chinese students might reduce the effects of light at night on sleep and circadian rhythm. When Japanese students were divided into those who lived alone and those who lived with their families, the effect of CCT was more pronounced among students who lived alone. This suggests that fewer social constraints for sleep habits make university students more susceptible to light at night. The relationship between the type of social constraints and the effect of night light on sleep needs to be examined in the future.

In this study, more than half of the Japanese and Chinese students used high CCT lighting at night. This result reflects the fact that it is common to use white and high CCT fluorescent lighting at night at home in some Asian countries including Japan and China. The percentage of students using high CCT lighting was significantly higher in Chinese students. The exact reason why white fluorescent lights are often used at home at night in Asia is not known. White fluorescent lighting began to become popular in Japan after the 1950s, and white fluorescent lighting becomes the preferred alternative to incandescent light in ordinary households. In the 1980s, fluorescent lamps with low CCT began to be sold, and it is thought that low CCT lighting has gradually become widespread in Japan. On the other hand, in China, low CCT fluorescent lights are not widely sold in stores and are not widely used at home. This may be one of the reasons for the differences between Japan and China. Currently, LED lighting is rapidly becoming widespread in both Japan and China. Since the CCT of LEDs can be easily adjusted, the lighting environment at home may be changing. Further research is needed because the lighting environment can be influenced by culture and technology.

For the survey of CCT light environments, we did not test its validity since it was a simple question. When the survey was conducted in 2014–2015, daylight color and warm color fluorescent lamps or incandescent lamps were generally used at home in Japan and China. Today, however, LED lighting with continuous color adjustment functions is widespread, and we may therefore need to be more careful about a survey of  CCT  of light using a questionnaire.

There were no significant correlations between illuminance level at night and sleep habits in this study. One reason may be that this study was a field study that included some uncontrolled factors affecting sleep habits other than the lighting condition at night. Although the purpose of this study was not to investigate the cause of differences in sleep habits of Chinese students and Japanese students, it is necessary to consider these factors that influence sleep habits. The low reliability of measurements of illuminance level using the pendant sensor is also a possible cause. For example, the problems with pendant-type sensors are that they may be hidden by clothing and the orientation of the sensor is not stable. Reliable measurements of illuminance level are needed to evaluate the relationships between individual sleep habits and illuminance levels at home. In addition, only 60 of the 447 Japanese and 60 of the 327 Chinese participants had their illuminance measured. Unfortunately, due to the small sample size in this study, we were also unable to examine the interaction between CCT and illuminance level.

Furthermore, the relationship between illuminance level and sleep habits may be complicated by individual differences in circadian photosensitivity [37–40]. In our previous studies, we found that polymorphisms in the melanopsin gene (*OPN4*) are associated with both the non-visual effects of light and the timing of sleep in Japanese university students [40, 41]. It would be interesting to compare the association between gene polymorphisms and sleep habits in both countries in the future.

In conclusion, the use of blue-enriched white light (high CCT light) at night is associated with delay of sleep timing in Japanese university students but not in Chinese university students. Although the effects of light at night on sleep timing and circadian rhythm may be complicated by other lifestyle factors depending on the country, use of low CCT light at home at night is recommended to prevent delay of sleep timing and the circadian rhythm.

## Supplementary Information

**Additional file 1: Figure S1.** The pictures used in the questionnaire in this study are not allowed to be shown due to copyright issues, but almost the same pictures are shown as additional files.

**Additional file 2: Table S1.** Demographic data and sleep habits in Japanese and Chinese students whose illuminance was measured.

## Data Availability

The datasets analyzed in this study are not publicly available due to a privacy policy but are available from the corresponding author on reasonable request.
