# Supplementary figures and images for: Is the use of high correlated color temperature light at night related to delay of sleep timing in university students? A cross-country study in Japan and China
Source: J Physiol Anthropol. 2021 Jun 8;40:7. doi: 10.1186/s40101-021-00257-x (PMC8188719; doi:10.1186/s40101-021-00257-x)

## Slide 1
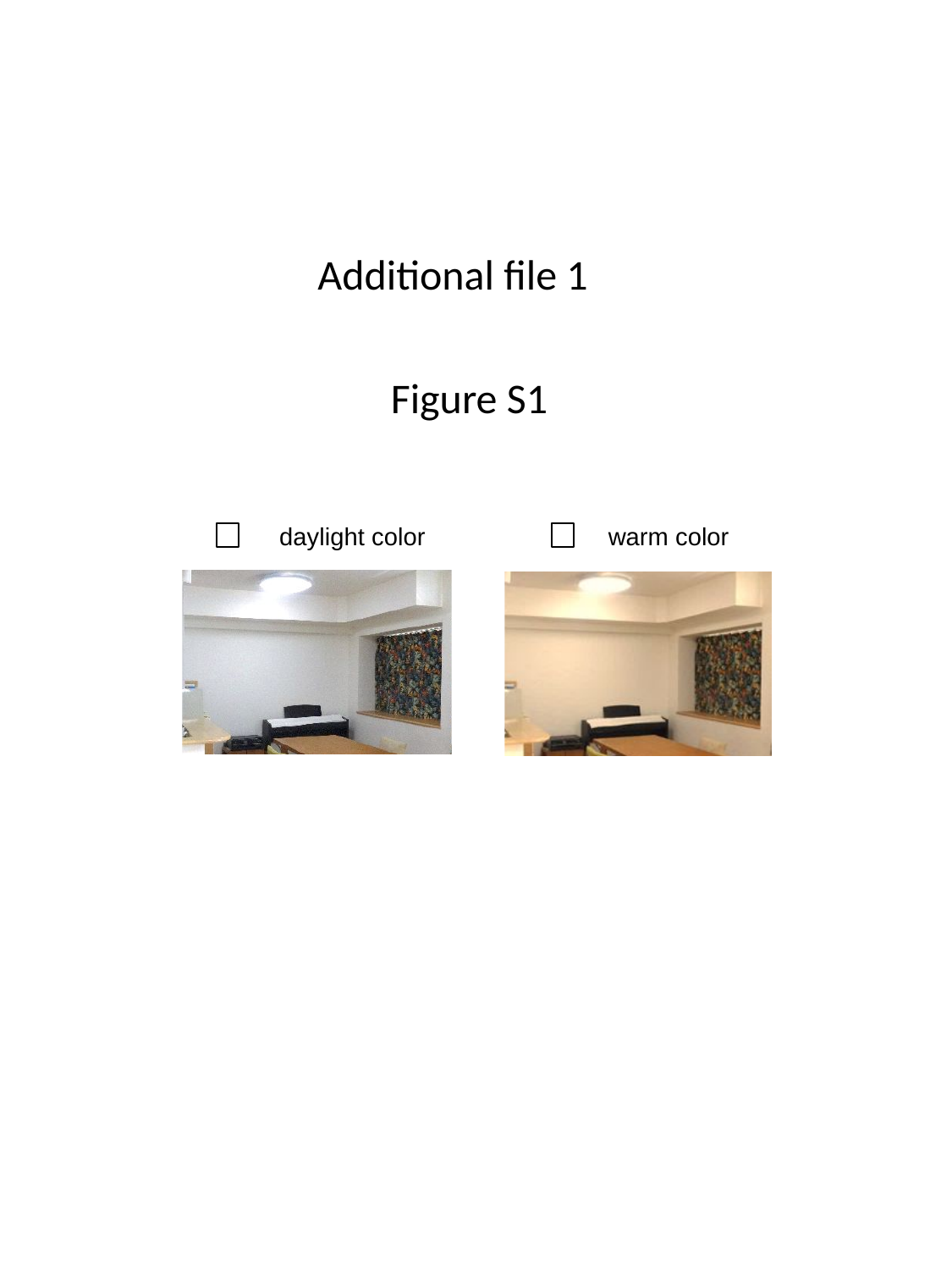

Additional file 1
Figure S1
daylight color
 warm color

Supplement: Supplementary file 1 — Additional file 1: Figure S1. The pictures used in the questionnaire in this study are not allowed to be shown due to copyright issues, but almost the same pictures are shown as additional files. [file 40101_2021_257_MOESM1_ESM.pptx]
