# Supplementary material for: Is the use of high correlated color temperature light at night related to delay of sleep timing in university students? A cross-country study in Japan and China
Source: J Physiol Anthropol. 2021 Jun 8;40:7. doi: 10.1186/s40101-021-00257-x (PMC8188719; doi:10.1186/s40101-021-00257-x)
Supplement: Supplementary file 2 — Additional file 2: Table S1. Demographic data and sleep habits in Japanese and Chinese students whose illuminance was measured. [file 40101_2021_257_MOESM2_ESM.docx]

Table S1. Demographic data and sleep habits in university students in Japan and China

|  | Japanese  (n=54) |  | Chinese  (n=58) |  |
| --- | --- | --- | --- | --- |
| Mean Age (years) | 22.5 (2.2) |  | 22.6 (2.2) | n.s. |
| Sex, %male | 51.9 |  | 50.04 | n.s. |
| **Sleep habits** (school days) |  |  |  |  |
| Bed time | 00:56 (1:05) |  | 24:15 (0:41) | *** |
| Sleep latency (min) | 15.0 (11.8) |  | 17.8 (13.0) | n.s. |
| Sleep onset time | 01:11 (1:05) |  | 00:33 (0:45) | *** |
| Wake time | 08:04 (1:05) |  | 07:48 (0:53) | n.s. |
| Midpoint of sleep | 04:38 (0:50) |  | 4:10 (0:40) | ** |
| Sleep period time (h) | 6.89 (1.39) |  | 7.25 (0.96) | n.s. |
| **Sleep habits** (free days) |  |  |  |  |
| Bed time | 01:24 (1:17) |  | 00:41 (0:46) | *** |
| Sleep latency (min) | 17.1 (16.`) |  | 15.3 (10.2) | n.s. |
| Sleep onset time | 01:41 (1:19) |  | 00:57 (0:50) | *** |
| Wake time | 09:34 (1:17) |  | 08:58 (1:06) | ** |
| Midpoint of sleep | 05:38 (1:04) |  | 4:57 (0:48) | *** |
| Sleep period time(h) | 7.88 (1.52) |  | 8.02 (1.09) | n.s. |
|  |  |  |  |  |
| Social jet lag (h) | 1.02 (0.77) |  | 0.76 (0.52) | * |
| Values are means and SD  Adjusted by age and sex **: p<0.01, ***: p<0.001, n.s: not significant | | | | |
